# A qualitative descriptive study of a novel nurse-led skin cancer screening model in rural Australia

**DOI:** 10.1186/s12913-022-08411-6

**Published:** 2022-08-10

**Authors:** Kristen Glenister, Sophie Witherspoon, Alan Crouch

**Affiliations:** 1 Department of Rural Health, ‘The Chalet’, University of Melbourne, Docker Street, Wangaratta 3677, Victoria, Australia; 2 Department of Rural Health, ‘Dunvegan’, University of Melbourne, 806 Mair Street, Ballarat, VIC 3350 Australia

**Keywords:** Rural, Skin cancer, Nurse-led, Programme logic model, Model

## Abstract

**Background:**

People residing in rural areas have higher rates of skin cancer and face barriers to accessing care. Models of skin cancer care addressing the specific needs of rural communities and overcoming specific challenges are required, but literature is scarce. This study aimed to describe the elements of a nurse-led skin cancer model in rural Victoria using qualitative methodology and programme logic to inform implementation and ongoing sustainability.

**Methods:**

Qualitative descriptive design. Semi-structured interviews were conducted with key stakeholders involved in the skin cancer model, namely health service executive management, clinical staff, and administration staff. Interviews were audio-recorded and transcribed verbatim. Transcripts were thematically analysed independently by two researchers before themes were compared and refined. A programme logic model was developed to organise themes into contextual elements, inputs, activities and anticipated outcomes; it was also used as a visual tool to aid discussions with key stakeholders. Member checking of the logic model occurred to verify interpretation. This programme logic model will be refined throughout the implementation phase, and again after three years of service delivery.

**Results:**

Eight stakeholders participated in interviews. Thematic analysis identified three major themes: the influence of the local rural context, the elements of the model, and “making it happen’. These major themes and accompanying sub-themes were mapped to the programme logic model by contextual elements (rural locale, health service access barriers, burden of disease), key inputs (promotion, human resources including appropriate nurse training and leadership) and ‘making it happen’ (governance including referral pathways, flexible and sustained funding, and partnerships). The anticipated outcomes identified include skin cancer care delivered locally, timely access, career development for nurses, and decreased skin cancer burden.

**Conclusion:**

An initiative that is place-based and community driven in response to consumer demand addresses key system barriers to earlier detection of skin cancers. It is anticipated to result in flow-on reductions in skin cancer disease burden. Programme logic was useful to both describe the initiative and as a visual tool for discussions, with the potential to inform wider health service efforts to address system barriers and bottlenecks.

**Supplementary Information:**

The online version contains supplementary material available at 10.1186/s12913-022-08411-6.

## Background

Australia has among the highest incidence rates of skin cancers globally, due in part to high ultraviolet (UV) radiation levels [1] and a predominantly fairer-skinned population [2]. Skin cancers, including basal cell carcinomas, squamous cell carcinomas, and melanomas, can be fatal and prompt management is crucial. For melanomas, prognosis is greatly improved by early detection [3]. This is especially important as melanoma mortality is significantly higher than that of non-melanoma skin cancers despite a much lower incidence [4]. Recent reports suggest that although the incidence of invasive melanoma among younger age groups may have reached a plateau or even in decline, the incidence among people aged 55 and older is rising. Targeted approaches to screening are required [5]. Accurate non-melanoma skin cancer incidence is difficult to ascertain, as data are incompletely collected, and most cases are managed in general practice [6].

### Rural areas

People residing in rural areas have higher rates of skin cancer and face barriers to accessing care [7]. Farmers and outdoor workers are exposed to three to eight times more UV radiation than indoor workers [8] and have a 60% higher mortality rate due to skin cancers than the general population [9]. Yet access to specialist dermatologist services in rural locations is severely restricted as 92% of dermatologists work in metropolitan areas [10]. Travelling to these metropolitan areas for specialist skin cancer treatment is associated with financial and time burdens [11] and extended waiting times for service access [12]. Extended wait times further contribute to later diagnosis and poorer outcomes. Rural Australians also experience individual level barriers to seeking or accessing skin cancer care such as a tendency to minimise the issue, a reluctance to complain and stoicism [12].

### Screening models

Federal policy supports well-designed screening initiatives [13] that emphasize patient recruitment through targeted service marketing strategies and a comprehensive quality framework. Australian-based screening includes initiatives led by general practitioners (GPs) [14] and nurses [15] with or without teledermatology [15]. These programs have been run out of community-based screening clinics [16] and workplaces [17]. The SkinWatch community-based programme was reported to be useful, acceptable and feasible [16]. Douglas and colleagues noted that there was room for improvement in workplace sun safety programmes, including provision of on site skin examinations and ongoing education for relevant employees [17]. Consumer self-examination with the naked eye or mobile teledermoscopy have also been trialled [18]. A recent systematic review highlighted that primary care provider clinical skin examination interventions are rarely evaluated for efficacy or effectiveness, and implementation methods tend to be poorly described [19].

### Nurse-led models of care

There is evidence that nurse-led approaches, including patient education in skin self-examination and clinical examination, dermoscopy and teledermatology, are fit-for-purpose in reducing skin cancer outcome disparities among high-risk groups with low access to GPs and specialist dermatologists [20]. Nurses in rural areas are well-positioned to deliver these models of care, as highlighted in submissions to the 2015 Standing Committee in Health Inquiry into Skin Cancer [9]. A pilot nurse-led model of skin cancer screening conducted in remote Western Australia was reported to be both professional and effective [15].

### Programme logic

Programme logic models are visual representations of programmes and include inputs, activities, and desired outcomes. These models are valuable as an integrated approach to describing and evaluating a new programme, involving stakeholders [21] and pointing to levers for change [22]. This approach has been used to describe educational programmes for primary prevention of skin cancer [23] and reporting on case studies of sun safety programmes for outdoor workers in Canada [24]. In these models, key elements include: skin cancer awareness, knowledge, beliefs and attitudes, and protective behaviours; all of which lead to decreased sunburns and skin cancers [23, 24].

## Methods

The aim of this study is to describe a newly developed nurse-led skin cancer screening and treatment model in rural Victoria initiated by a rural health service in response to health service stakeholders and community demand. This region has high melanoma incidence and mortality rates [25]. The study design (qualitative, descriptive) was chosen to gain a deeper understanding of the model’s elements, and to determine how to best support implementation, and sustainability. communicate learnings that may be useful to other health services exploring approaches to addressing similar challenges.

### Setting

The population in the region is geographically dispersed and has experienced long-standing GP workforce shortages. There are high proportions of older people and farmers, two groups at higher risk of skin cancer. West Wimmera Health Service provides services to a catchment of 16 000 people with five small rural hospitals (2–35 beds) and the following facilities: residential aged care, disability and community health. The health service catchment area spans 22 000 square kilometres [26]. A series of preliminary pilot clinics (four clinics between August 2018 and August 2019 with 225 appointments) were conducted by a nurse practitioner with specialist training in skin cancer identification at West Wimmera Health Service, and were oversubscribed. The proposed ongoing skin cancer initiative involved running screening clinics led by a group of local nurses and nurse practitioners within the West Wimmera Health Service Health Service catchment area. The nurses and nurse practitioners will received specialist skin cancer screening and dermoscopy training followed by a period of shadowing or mentoring with a qualified skin cancer specialist nurse practitioner. Clinics will be advertised using mainstream and social media. Clinics will be held regularly in the cooler five to six months of the year, as lesions can be more challenging to identify on tanned skin during the warmer months. Patients with suspicious lesions will be referred to local GPs as appropriate or to dermatologists in Ballarat, Horsham, or Hamilton. The evaluation component of the project is multi-stage. It includes (a) the qualitative, exploratory, descriptive study, (b) a quantitative assessment of the impact of the model on health outcomes and referrals, including patient experience of the clinics, and (c) an updated description of the model and impact assessment after three years of service delivery.

### Participant characteristics

Key stakeholders were involved in the development and delivery of the preliminary pilot clinics. Potential participants were identified by the West Wimmera Health Service Management Team, including health service administration professionals, leadership team members, health practitioners undertaking training and clinical partners. A large proportion of the project stakeholders were identified, and all invited stakeholders participated in interviews.

### Participant recruitment and semi-structured interviews

Purposive recruitment was used to select the participants. Potential participants were contacted by a researcher (Author 3) via email, telephone, or both. Participants were then invited to undergo in a semi-structured interview. They were aware of the skin cancer initiative and the aims of the research study. Interviews were conducted by a single researcher (either Author 1 or Author 3) via telephone, videoconferencing, or in person at the participants’ workplace. No other people were present at the interview. Interviews were audio-recorded and transcribed verbatim. Authors 1 and 3 were rural health academics with deep interest in health equity, and residents of rural Victoria. Author 1 is a white, middle-aged female with a science, chronic disease, and health service research background. Author 1 had no prior relationship with any of the participants. Author 3 is a semi-retired male with a health programme research and global health background. Author 3 had initial contact with health service management through the consumer advisory group of the regional integrated cancer service. For semi-structured interview questions, please refer to Additional file 1. Interviews were conducted between October 2020 and August 2021, at a time when five nurses had received training, but were not yet running solo clinics due in part to disruptions resulting from the COVID-19 pandemic.

### Ethics

Ethics approval was granted by the Ballarat Health Service and St. John of God Healthcare Human Research Ethics Committee and University of Melbourne Human Ethics Committee. All methods were carried out in accordance with relevant guidelines and regulations. Informed consent was provided by each participant prior to interviews.

### Qualitative analysis and programme logic model

Transcripts were thematically analysed by two independent researchers to achieve an in-depth description of the nurse-led model, its context, and anticipated outcomes. Thematic analysis followed the six steps described by Braun and Clarke [27]: namely familiarization with the data, coding according to the study aim, identification of common patterns among codes and arrangement into potential themes, review of themes, refinement of themes, and finalisation of results. After deliberation, the authors refined a programme logical template that arranged the identified themes into context, input, activities, outputs, and outcomes (direct, indirect, final) [22]. Immediate outcomes were defined as those within direct control of the health service and clearly attributed to the outputs. In contrast, intermediate outcomes, particularly final outcomes, were under less control of the health service, as described by Watson et al. [22]. Member checking of the programme logic model was conducted after completion of the interviews, during discussions with key stakeholders in August 2021. Member checking occurredto ensure an accurate reflection of participant views, enable the addition of data where necessary and verify the interpretation of findings. The questions posed to the meeting attendees were ‘does this programme logic model capture your experience of the clinics and its potential impacts’ and ‘are there missing elements of the programme logic model’. This study followed the Consolidated Criteria for reporting Qualitative research (COREQ) guidelines, in which member checking is recognized as a method of rigor [28].

## Results

Eight interviews were conducted, three with people in health service management roles, three skin cancer nurses/nurse practitioners, one health promotion officer, and one general medical practitioner, as per Table 1. Of the potential participants invited to participate, none refused, nor did any participants drop out of the study. Interviews ran for an average of 19 min (ranging from 15 to 43 min). Three major themes were identified, namely the local rural context, the elements of the model and “making it happen’. These three themes encompassed eight sub-themes (farming/weather, access to health services, burden of disease, promotion of services, staff development, governance, funding, and partnerships). The themes and sub-themes, along with illustrative quotes (denoted MGT for management participants, N for nurse or nurse practitioner participants, HP for health promotion participants, and GP for general medical practitioner participants) are outlined in Table 2.

**Table 1:** Description of participants

Participant group	Number of participants
Health service management	3
Nurse, nurse practitioner	3
Health promotion	1
General medical practitioner	1

**Table 2:** Themes, sub-themes and illustrative quotes

Theme	Sub-themes	Quote
Local, rural context	Farming, weather	…but we are a traditional farming community…and you know people in these areas haven't been big in covering up as much as they would be expected, everyone expects people to do these days **MGT3**
	Access to health services (distance, workforce shortages, waiting time)	…the barriers for people are just the general tyranny of distance… if they had to go to Ballarat it's a 300 km drive, it's an all day trip **MGT3** Horsham has a long waiting list, because that area covers the area all around the Wimmera…Their waiting time could be one and a half months or two months **GP**
	Burden-of-disease; later diagnosis; poorer outcomes; awareness of skin cancer risk; demand for skin cancer screening and treatment	Various cancers are one of our biggest burden-of-disease issues, or one of our most amenable [to intervention] burden of disease issues in our sub-region, particularly skin cancer screening **HP** …cancer awareness and cancer prevention is very much part of that broader health promotion strategy looking at the burden of disease and the major environmental and structural factors **MGT1** So a sense, a huge sense, of need and that actually people are increasingly aware now and if there's a clinic there they want to go. We haven't got to drag people along kicking and screaming **MGT1** In our catchment area again, because we're fairly rural, remote and rural, we know, or the stats tell us that people who do get any type of cancer generally have worse outcomes than in the city. They have a higher chance of getting any types, most types of cancer than people in the city and if they do get it their survival rates aren't as great **MGT3** So, we put out the advert (for skin cancer checks), and of course, we had 100 people ringing up, we filled the day of about 30 and then had 100 on the waiting list, we actually did deliver all those skin cancer checks over the course of a few months **N2**
Elements of the model	Promotion of the service	Now, for a large part, that [promotion of the service] really has relied over the last few months in getting information out on social media…. of course our staff is a pretty significant proportion of the general population, if we get them talking about it, hopefully the trickledown effects touches almost every household **(HP)**
	Staff development. Getting the right people trained with optimal training and mentoring and ensuring a smooth path to solo practice	…but there is no way I would be confident to assess a spot, diagnose a spot and treat a spot with [just] that training… **N1** … one that actually stayed with me and came two days [of scheduled skin cancer screening clinics]…said that was really good…to know what you were looking for… **N1** I think that they would really need probably a full week…with me doing 50 consults a day…would be the bare minimum in order to maintain some sort of proficiency in dermoscopy **N1** I think we had about 13 expressions of interest, but we only had funding for, originally, one position, but then management/exec decided that they'd extend this to offer it to five persons, ….we selected five staff that would cover a broad area in our nine sites **N2** It (the dermoscopy training) was not really what I expected. So it was targeted very directly, very strongly towards GPs. So there were five of us nurses that all went from West Wimmera Health Services and they were the only nurses in the entire room. I feel probably like it wasn't the best option in terms of courses that we could have done because they would give us sort of the basic knowledge but then they would just flash all these pictures on the screen and say would you excise this or not excise this?…we can't excise…Of the five of us that completed the course, only two of us actually passed the exam **N3**
Making it happen	Governance (billing, referral pathways, data management)	Efficiency and the timeliness, well, the timeliness will be around that before we actually run one of these clinics, we need to have our referral pathway, everything needs to be sorted. So, when we start, we start properly. Because that's been my biggest concern that we are going to start—excuse the language—this half-arsed thing, that we haven't actually worked out what we're doing **MGT2**
	Funding (stability and flexibility)	Yeah, well I think that was the other driver for being involved in the project, was that currently our funding model allows for it. So we get funded, block funded through Community and Women's Health and all our allied health and community health nursing is funded through that. So currently our community health nurses can claim—they'll take pathology and do consults and they can claim that time against this funding model. When we looked at it we really thought there's no reason why this would be any different **MGT1**
	Partnership, collaboration, stakeholders	If our local GPs know that, what is the system, that will help them understand, that you know, we can all work as a team **GP** I think probably the biggest internal factor that we have is motivating the person who is supposed to be implementing all of this sort of stuff to happen **N3** Wimmera Primary Care Partnership, they had some funding to do a health literacy project in Hindmarsh and Yarriambiack shires…we actually put out some surveys, about what residents in both those areas would like to see, what came to light, there was a particular need for men's health and skin checks **N2**

### Local, rural context

The rural context was relevant to consider in terms of:

- Exposure to UV radiation
- At-risk groups of people, including older people, men, and farmers
- Restricted access to skin cancer services
- Recognition of poor health outcomes due to late diagnosis

#### Farming, weather

Participants spoke of farmers’ difficulties with reliably finding time to make and attend multiple appointments, needing to work around essential periods on the farm (eg. harvest), and historically being less “sun smart’.

- Farmers, like they are busy and don’t get time...because this idea of going to the GP, and then getting an appointment with a skin cancer clinic is just, you know, we’ll do that, we’ll do that when we have time, after harvest maybe (GP)

Participants described previous skin cancer screening initiatives, including taking bus-loads of farmers hundreds of kilometers to the neighboring state’s capital city, orincorporating them into farmer health checks, including at field days.

- Historically, buses used to run from these areas and there was often funding for farmers to catch these buses all the way to Adelaide to have their skin checks. It actually meant that these people were leaving at three and four o’clock in the morning and that was their whole day taken (N2)

#### Access to health services

Participants outlined the barriers faced by local people when accessing health services for skin cancer screening, including out-of-pocket costs, extended travel distances, and long waiting times due in part to persistent health workforce shortages. These barriers potentially led to people delaying access, or even not accessing services at all. This was potentially compounded for some groups of people, including older people.

- I mean the barriers for people are just the general tyranny of distance ones. Where someone might, if they had to go to Ballarat, it’s a 300k drive, it’s an all day trip. You might get there and you might be like if you’re late or the doctor or whoever you’re seeing might be half and hour late, it’s a massive day. Typically, these people are a bit older, so that adds to it all (MGT3)

#### Burden of disease

Participants spoke of links between rurality, late diagnosis, and poorer health outcomes but also discussed this issue as being amenable to change. One participant spoke of the COVID-19 pandemic delaying screening and likely having ramifications for diagnosis of new lesions. Several participants spoke of the loss of a young parent due to melanoma.

- Various cancers are one of our biggest burden of disease issues, or one of our most amenable burden of disease issues in our sub-region, particularly skin cancer screening (HP).

### Elements of the model

Participants spoke of an ideal model being locally run, ongoing, regular, and in-person. Participants spoke of the community awareness of skin cancer and demand for screening, resulting in the oversubscription of initial clinics. Participants also highlighted the advantages of the model being nurse-led. One participant (N2) spoke of a healthy eating and exercise initiative that was successful due to it being local and widely advertised, features that could be replicated in the skin cancer screening model.

- A lot of people are very comfortable seeing a nurse rather than going to a GP. We have Pap smear clinics and other like screening clinics run by nurses. So it’s sort of the model that we’re used to and that we know works quite well (MGT1).

#### Promotion of services

Participants spoke of awareness of the service being raised by mainstream media, social media, word of mouth, and a local podcast.

- We know that when we have put things about it on Facebook, the sessions that we have had get quickly taken up…you do see generally daughters and sons or children of people who probably need a test, an old farmer perhaps, or grandpa or grandad. You see them sharing that with their, might be friends or someone on Facebook (MGT3).

#### Staff development

Nurses spoke of the opportunity to pursue skin cancer screening education/training as professional development. Training issues were also raised, including that an adequate number of the right people needed to be trained and the training required to be tailored to nurses. In addition, training needed to be followed by a substantial period of shadowing to enable them to reach an adequate level of confidence and competence to undertake solo clinics. Ongoing ‘refresher' training would also be necessary to ensure best practice over time.

- It [the training] was not what expected. So it was targeted very directly, very strongly toward GPs. So there were five of us nurses that all went from West Wimmera Health Services and they were the only nurses in the entire room. So it was...I feel probably like it wasn't the best option in terms of courses that we could have done, because they would just flash all these pictures on the screen and say 'would you excise this or nor excise this?' I thought well, that's not really- we can't excise (N3)

### 'Making it happen'

Participants spoke of the important aspects of developing and implementing the skin cancer screening model, including partnerships with key stakeholders, clarifying expectations of key stakeholders, governance issues (including policies, procedures and pathways), and funding. Participants recognised the importance of embedding a new model and the timeframes involved.

- So I think the thing about embedding it is being committed, recognising that there’s not a quick fix and it takes a long time to embed things (MGT1).

#### Governance

Participants spoke about the need for clear policies and procedures, including referral pathways for patients with identified lesions. These pathways could build upon existing approaches and relationships. The potential concern that GPs would not be supportive of the model was allayed by several participants who pointed out that a nurse-led clinic would streamline skin cancer management and take some of the burden off a historically stretched GP workforce.

- I think the GPs need to take some ownership of it [the nurse-led model] in terms of shared ownership, I should say. The GPs can’t do it all themselves...I do skin checks in all of those towns [Nhill, Warracknabeal, Ouyen, Hopetoun] and every town the GPs say the same. 'We can’t do it all, and we’re happy that if you’re coming to help out and you’re referring appropriately then you’re doing a good service’. GPs need to work with us rather than say 'well yeah, I’ll just manage anything skin spot that comes through the door’ (N1)

#### Funding

Participants spoke of the role that flexible, ongoing funding played in being able to consider a new place-based model for skin cancer screening.

- I think that was the other driver [in addition to local burden of disease] for being involved in the project was that currently our funding model allows for it. So we get funded, block funding through Community and Women’s Health and all our allied health and community health nursing is funded through that. So it’s a broad criteria programme around meeting the needs of the community and delivering primary community health nursing services. (MGT1).

#### Partnerships

Participants spoke of existing partnerships between West Wimmera health service staff and nurse practitioners, dermatologists and GPs, and highlighted the value of working as a team. A health promotion staff member spoke of a unique community outreach approach that has been successful in engaging with new community members regarding their health and awareness of local services, which will be utilised in the future in relation to skin cancer screening.

- If our local GPs know that what is the system, that will help them understand that you know, we can all work as a team (GP).

**Fig. 1:**
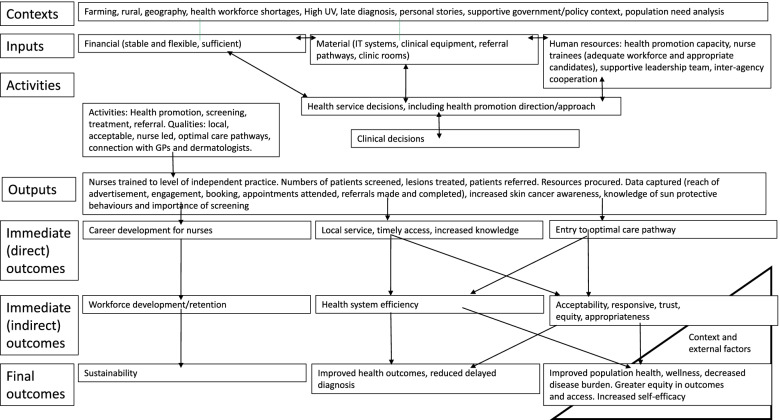
Qualitative results arranged as a programme logic model

The themes and sub-themes were organised into context, input, activities, outputs, outcomes (direct, indirect, final) with a programme logic template [22]. The resultant programme logic model is described below (Fig. 1).

## Discussion

Successful implementation and sustainability of the nurse-led skin cancer care model requires and in-depth understanding of the elements comprising the model, its context, and the anticipated outcomes. An exploratory, descriptive study design was valuable in gaining this level of understanding. Description of the skin cancer model incorporated contextual factors related to the rural locale, the need for adequately trained and supported nurses and the importance of flexible, sustainable funding, clear governance structures, and partnerships. A similar programme logic model approach has been used to address sun safety among Canadian outdoor workers [24] and to underpin a review of skin cancer education interventions for primary prevention of skin cancer [23].

The described model of care had foundations in effective relationships and flexible funding which enabled innovative solutions to meet local needs. The described model of care overcame many of the access barriers that local people currently face when accessing skin cancer screening, including distance, out-of-pocket cost, and extended waiting times exacerbated by workforce shortages. The model of care is nurse-led, building upon other nurse-led models of care which have been shown to be acceptable to patients (eg. sexual health care and chronic disease management [29]). The crucial role of partnerships and effective leadership was highlighted in embedding the model in the long term. A study in regional Queensland using store-and-forward teledermatology reported that residual issues remained with inadequate image quality (16% of referrals) and remuneration for the health professionals involved [30]. Another study in regional Queensland reported that an initiative including education and screening was acceptable and feasible, and supported by local doctors as a way of assisting with work capacity limitations [16] although it is unclear whether the initiative was sustained long-term. A pilot study in remote Western Australia involved screening of 54 patients over four days by nurses, with 6 malignant melanomas excised and 15 non-melanoma lesions treated [15].

The application of a programme logic approach was useful in: 1) identifying current gaps in the model (for example the need for clarification of referral pathways); 2) demonstrating the complexity and interconnectivity of factors within the model (for example older people or farmers being at higher risk of skin cancer, but potentially facing barriers in accessing care, such as extended travel times or time away from the farm), and 3) points for evaluating the programme (for example numbers of nurses trained to the level of solo practice or numbers of people accessing screening). In other studies that used programme logic, an evaluation of patient experience conducted in six wards in United Kingdom hospitals reported that facilitation skills, feedback and team performance influenced patient experiences and health outcomes [31]. In a study describing tobacco control measures, implementing a new policy influenced attitudes, beliefs, and behaviours, which in turn influenced health outcomes [32]. Description of a new model at an early stage of implementation is unusual, and addresses the issue that methods of skin cancer initiative implementation are rarely described, as has been raised previously [19]. Member checking with the key stakeholders utilised the logic model as a visual aid. At the member checking meeting, valuable discussions occurred re the importance of health promotion activities, meeting the needs of under-served populations and breadth of referral pathways.

The model was initiated in response to recognised local need, and oversubscription to early clinics demonstrated community awareness of the issue and demand for the service. The model had elements of co-production insofar as a range of stakeholders were involved in the design of the initiative and shared decision-making, which has been demonstrated to be beneficial in terms of engagement, integration of services, adaptation, and sustainability, particularly in rural areas [33]. The place-based nature of the initiative addresses issues related to rurality and the unique nature of rural communities [34].

### Limitations

The findings from this study may not be applicable to other rural contexts. However, the key elements of this innovative, community-driven, nurse-led model identified here are likely to be useful in informing other nurse-led and rural models of care. The patient perspective is currently absent from this study and will instead be included in the next stages. Sub-group analysis of the logic model, for example for smaller centres within the health service catchment, or for people who have never engaged with skin cancer screening, is yet to be undertaken.

### Future directions

This study has been designed as a multi-stage study, and the programme logic model will be refined after three years of service delivery and will be used to underpin the quantitative assessment of impact of the initiative on health outcomes and referrals. Quantitative data will be collected to assess numbers of patients screening, lesions detected and treated, proportions of early and late-stage lesions and type as well as data related to nursing staff training, mentoring, and service delivery.

## Conclusion

Novel, rural targeted models of care for skin cancer are uncommon, and this qualitative, descriptive study provides insight into the elements of one such example. A nurse-led, targeted approach to skin cancer screening for at-risk rural populations, driven by high local burden of disease, strong community demand, underpinned by innovative health service management and a flexible public health funding mechanism, has the potential to reduce skin cancer morbidity and mortality in rural Victorian settings.

## Supplementary Information

**Additional file 1. **Semi-structured interview questions.

## Data Availability

Due to potential identifiability of participants, data are not available.

## Abbreviations

COREQ: Consolidated Criteria for reporting Qualitative research
COVID-19: Coronavirus disease of 2019
GP: General practitioner
UV: Ultraviolet
