# Supplementary material for: A qualitative descriptive study of a novel nurse-led skin cancer screening model in rural Australia
Source: BMC Health Serv Res. 2022 Aug 10;22:1019. doi: 10.1186/s12913-022-08411-6 (PMC9365213; doi:10.1186/s12913-022-08411-6)
Supplement: Supplementary file 1 — Additional file 1. Semi-structured interview questions. [file 12913_2022_8411_MOESM1_ESM.docx]

Additional file: Appendix 1. Semi-structured interview questions.

**Nursing Staff**

1. Could you briefly describe your nursing career in general and the reasons you became interested in skin cancer screening in particular?

2. What specific training have you undertaken to enable you to conduct skin cancer screening? a. Prompt: Dermoscopy? Dermatology? Skin lesion identification? b. Prompt: Mentorship in skin cancer screening? c. Prompt: Other?

3. How did this training prepare you for clinical practice in skin cancer screening?

4. What, if any, further training opportunities would be of use to you as you continue your skin cancer screening activities?

5. Please describe your level of confidence in identifying the major skin cancer types: melanoma; basal cell carcinoma; squamous cell carcinoma; actinic keratosis?

6. How does your rural health service organise skin cancer screening? a. Prompt: Episodic pre-booked clinics? Where? What frequency? b. Prompt: Opportunistic screening? How monitored? c. Prompt: Referral pathways d. Other?

7. How do you conduct a skin cancer screening consultation? a. Prompt: Total body screen? b. Prompt: Patient-identified lesion(s)? c. Prompt: Other?

8. What do you do when you identify a suspicious lesion? a. Prompt: Treatment? b. Prompt: Referral to a general medical practitioner? c. Prompt: Bottle necks to on referral of suspicious lesions? d. Prompt: Other action?

9. What is the time to further treatment following referral to a general medical practitioner in your rural setting?

10. How do you follow up on patients following treatment or referral to a general medical practitioner? a. Prompt: Routine recall system? b. Prompt: Other?

11. What internal and/or external factors, if any, constrain the embedding of the skin cancer screening function into routine primary care in your health service?

12. What support do you have from your health service management to develop and extend the skin cancer screening service?

13. How could efforts in in skin cancer screening be further expanded within your communities?

14. What other comment would you like to make?

**Nurse Practitioner (Mentor)**

1. What in your view is a suitable period for mentorship in skin cancer screening for newly trained and qualified specialist nurses?

2. What continuing professional education would you recommend for specialist skin cancer nurses?

3. What processes do you recommend to the specialist nurses regarding collaborative engagement with local general practitioners and/or relevant resident or visiting specialists

4. What, in your view, are the critical factors contributing to retention by rural health services of specialist nurses trained in skin cancer screening?

**Health Service Medical Staff / Private General Practice Staff**

1. What capacity is there within the local rural hospital medical staff / private rural general practitioner workforce to absorb specialist nurse-led skin cancer screening referrals?

2. To what degree are local medical practitioners supported for additional professional training in skin cancer treatment, where required?

3. To what degree is biopsy / excision of suspicious lesions on the day(s) of the specialist nurse-led clinics a realistic option in your local rural setting?

4. How difficult is access in your local rural setting to specialist medical / surgical services where these are indicated in the treatment of referred skin cancer patients?

5. What are your expectations of the specialist nurse-led skin cancer screening initiative in terms of reaching under-screened or never-screened populations and individuals?

6. What other comment do you wish to make?

**Health Promotion Staff**

1. What formal or informal agreements do you have with the clinical services providing skin cancer screening in your rural area?

2. What approaches have you / your agency taken to attract under-screened and never-screened populations and persons to organised skin cancer screening events in your rural health district? a. Prompts: Public skin cancer information programs; specific screening event campaigns; role of social media; other approaches?

3. How have you determined the degree to which these approaches have been effective and what factors underlie this effectiveness? Please give some examples.

4. How will you determine that all at-risk groups in your rural setting have been reached by skin cancer screening messages and have had a realistic opportunity to access skin cancer screening? a. Prompts: which populations; clinic locations; demand measures

5. What sources of renewable funding do you have at your disposal to promote skin cancer screening?

6. What other comments would you like to make?

**Health Service Management Staff**

1. To what degree do responses to the burden of skin cancer disease feature in your in your health service strategic planning? a. What was your priority in this context and what factors influenced your decision? b. What led your health service to explore a c. nurse led skin cancer screening approach? d. How do you plan to embed nurse-led skin cancer screening into the routine primary care services delivered across all sites of your rural health service?

2. What is the capacity for referral of skin cancer patients requiring further treatment following screening to general medical practitioners and medical specialists in your local context?

3. What local factors influence the efficiency, timeliness and effectiveness of referrals?

4. What funding, cost recovery and other revenue streams are available to your health service to strengthen the financial sustainability of skin cancer screening across all sites of your rural health service?

5. What other comments would you like to make?
